# Serum lactate upon emergency department arrival as a predictor of 30-day in-hospital mortality in an unselected population

**DOI:** 10.1371/journal.pone.0190519

**Published:** 2018-01-02

**Authors:** Yong Joo Park, Dong Hoon Kim, Seong Chun Kim, Tae Yun Kim, Changwoo Kang, Soo Hoon Lee, Jin Hee Jeong, Sang Bong Lee, Daesung Lim

**Affiliations:** 1 Department of Emergency Medicine, Gyeongsang National University School of Medicine and Gyeongsang National University Changwon Hospital, Changwon, Gyeongsangnam, Republic of Korea; 2 Department of Emergency Medicine, Gyeongsang National University School of Medicine, Jinju, Gyeongsangnam, Republic of Korea; 3 Gyeongsang Institute of Health Sciences, Gyeongsang National University School of Medicine, Jinju, Gyeongsangnam, Republic of Korea; Azienda Ospedaliero Universitaria Careggi, ITALY

## Abstract

**Background:**

Despite of numerous evidences that elevated serum lactate levels were associated with unfavorable outcomes, there have been no study demonstrated an optimal cutoff of serum lactate in unselected patients. This study was aimed to evaluate the prognostic property of lactate, and to identify a cutoff of serum lactate level for predicting 30-day in-hospital mortality among unselected patients presenting to the emergency department (ED).

**Methods:**

We performed a retrospective observational study from January 2010 to December 2016. 61,151 patients were used for propensity score analysis after exclusion. 14,015 patients who underwent lactate test at ED arrival were enrolled for final analysis.

**Results:**

The average treatment effect (ATE) of carrying out a lactate test on 30-day in-hospital mortality was 0.53% (adjusted odds ratio (OR) = 1.013, *p* = 0.19; 95% confidence interval (CI), 0.997–1.013). Adjusted OR of serum lactate calculated from multivariable analysis was 1.09 (*p* < 0.001; 95% CI, 1.07–1.10). The area under a ROC curve (AUC) of serum lactate was 0.711 (*p* < 0.001; 95% CI, 0.703–0.718). The sensitivity, specificity, and positive and negative predictive values for the cutoff > 2.6 mmol/L were 56.7%, 74.3%, 20.8%, and 93.5%, respectively. Mortality of the high-lactate group (> 2.6 mmol/L) was significantly higher than that of the low lactate group (≤ 2.6 mmol/L) (20.8% vs. 6.5%, difference = 14.3%, *p* < 0.01; 95% CI, 13.0% - 15.7%).

**Conclusions:**

A serum lactate level > 2.6 mmol/L predicted 30-day in-hospital mortality in unselected patients who arrived to the ED and were admitted to the hospital. Additionally, serum lactate test in the ED could be an effective screening method for identifying low risk patients.

## Introduction

Hyperlactatemia has been shown to be related to higher mortality in selected patients admitted in the intensive care units (ICUs) with specific conditions such as sepsis, trauma, or critical illness [1, 2]. In the emergency department (ED) setting, several studies reported similar results in patients with specific diagnoses [3, 4]. However, the limited study populations lead to narrow applicability of serum lactate. Many previous studies evaluated the relationship between serum lactate level and mortality in ED patients with specific diagnoses [5, 6], however, studies of unselected patients were few [7, 8]. Because serum lactate tests have not been established as part of a routine work up in unselected ED patients, selection bias was unavoidable in studies that evaluated the relationship between serum lactate level and mortality in patients in the ED setting. To overcome this limitation, a recent study included a control group of patients in whom lactate was not measured; the study showed hyperlactatemia was associated with higher mortality. However, this study also had some limitations such as a relatively small (n = 600) and restricted (ED patients with internal disease) study population [9].

Another issue regarding the feasibility of serum lactate is determination of the cutoff value. Despite a value of ≥ 4.0 mmol/L being recommended for prompt resuscitation in current international guidelines for the management of sepsis and septic shock [10], a recent study in patients with septic shock in ICU setting reported a value of 2.5 mmol/L as a cutoff in predicting mortality among patients with severe sepsis and septic shock [11]. Besides, other previous studies have shown that lactate levels lower than 4.0 mmol/L were associated with poor outcome [12, 13]. More recently, combination of the National Early Warning Score (NEWS) and lactate has been studied as a triage tool for predicting outcomes of ED patients [14]. The study showed lactate provided additional predictive value to the NEWS and NEWS plus lactate were superior to the NEWS alone in predicting unfavorable outcomes.

Despite these abundant studies suggested that lactate may play an important role in detection of patients with high risk for poor outcome in various clinical situations, there have been no studies demonstrating whether lactate can be used as a prognostic marker in truly unselected ED population.

The aim of this study was (1) to evaluate the prognostic property of lactate, (2) to identify a cutoff value of serum lactate in predicting in-hospital mortality among unselected patients presenting to the ED, and (3) to assess the outcomes in patients with hyperlactatemia defined by the determined cutoff value.

## Materials and methods

### Study design

This was a single center retrospective hospital-based cohort study that analyzed patients presenting to the ED of the Gyeongsang National University Hospital, which is a tertiary referral hospital located in the south-central region of the Republic of Korea. Gyeongsang National University institutional review board approved this study with the exemption of informed consent because of the retrospective nature of the analysis.

### Study setting and participants

All patients arriving at the ED must be enrolled in the National Emergency Department Information System (NEDIS) of Korea. The input data are organized using the standard NEDIS registry format in the electronic medical records (EMR) of the hospital and sent to the NEDIS server. The NEDIS is a national database that is prepared by 146 emergency medical centers and managed by a government-funded national ED control agency [15, 16]. Initial data from patients are entered by triage nurses and duty doctors at ED arrival: physiologic parameters at ED arrival, symptoms, and diagnosis. Basic demographic and temporal information, treatment details including drugs and procedures, outcomes, and other information are created from the EMR and transferred to the registry. Validity of all data is checked by function modules within the EMR system before the data is saved.

Consecutive patients who presented to the ED between January 2010 and December 2016 were enrolled. Patients were included if they were ≥ 18 years of age, admitted to the hospital after ED management, and had lactate result in the first three hours after ED arrival. We excluded patients who were transferred to other facilities after admission, discharged with no hope of recovery, or left the hospital against medical advice, because the final outcomes of these patients could not be determined.

The annual ED census of the hospital during the study period ranged from 32,000 to 35,000 patients.

### Study protocol and measurements

Data were extracted from the EMR system of the hospital. Demography (age and sex), patient′s categorization (disease stemming from medical illness or injury from external cause), physiologic parameters (mental status, systolic blood pressure, heart rate, respiratory rate, body temperature, and oxygen saturation (SpO_2_)), laboratory results (lactate, white blood cell count (WBC), hemoglobin, platelet, international normalized ratio of prothrombin time (PTinr), glucose, creatinine, bilirubin, c-reactive protein (CRP), and base deficit), time variables (date of ED arrival, death, and discharge), and final outcome (discharge, transfer, death, or other) were collected. The patients were classified into subgroups for further analysis: diseased or injured by patient category at ED arrival; patients with suspected infection (antibiotic use within 24 hours) or without infection.

We analyzed the first laboratory results within three hours after ED arrival. The serum lactate tests were embedded in the point-of-care testing (POCT) module in the ED, and the results were reported within 5 minutes after blood sampling. The POCT module included arterial blood gas, electrolytes, complete blood count, and lactate. Blood samples were mostly collected from the radial or femoral artery. Other blood tests were ordered as routine work-up. Final discharge results were extracted from the discharge summary in the EMR system. Primary outcome was 30-day in-hospital mortality.

### Data analysis

Because of the retrospective nature of the study and concern of selection bias, we evaluated whether execution of the serum lactate test was associated with the outcome (30-day in-hospital mortality) using propensity score analysis. Serum lactate was not performed as a routine test in patients who presented to our ED. Physicians tended to order the test when patients appeared to be in serious condition, such as worsening physiological parameters, decreased mentality, lower SpO_2_, and significant results from other routine blood tests. Additionally, because our POCT module comprised blood gas, electrolytes, complete blood count, and lactate, physicians also tended to order the POCT (with the serum lactate test) as a follow-up test. The propensity of carrying out a lactate test were estimated using a logistic regression with all available information before the physician decided to order the test. Average treatment effect (ATE) of execution of lactate test on 30-day in-hospital mortality was calculated using propensity score matching analysis with the inverse probability weights method. We hypothesized that selection bias would be minimal if the ATE was less than statistically significant level.

We used multivariate imputation with chained equation (MICE) to impute all missing values [17]. The number of multiple imputations should be increased as the fraction of missing information increases. Most researchers accepted the rule that the number of imputations should reach the percentage of missing cases (e.g., at least 10 iterations for 10% missing data) [18, 19]. In our data set, base deficit was the most frequently missing value (6.4%), followed by the value of CRP (3.0%), SpO_2_ (1.3%), creatinine (1.2%), complete blood cell count (0.3%), and physiologic parameters (0.1~0.3%) (Table 1). To minimize additional variation due to the estimation of the missing data, we conducted 20 imputations considering the variable with the largest missing fraction (6.4%, base deficit). An important premise for multiple imputation analysis is that the missing data mechanism depends on the variables in the same dataset (missing at random). In our study, missing data mostly occurred in laboratory results. The physicians’ decision to order a specific blood test mainly depended on initial clinical findings such as demographic characteristics and physiologic parameters; therefore, we assumed that our data were missing at random. Multiple imputation with the MICE method by predictive mean matching was performed to create 20 multiply-imputed datasets. Univariable and multivariable logistic regression analysis were performed for all the demographic, physiological, and biochemical variables in each imputed dataset, and the results were combined. Every combination of steps for the estimated results from the imputed data followed the Rubin’s rule [20]. After verification of the adjusted odds ratio (OR) of serum lactate, receiver operating characteristics (ROC) curve was constructed. The lactate cutoff was chosen as the maximum value on Youden’s index calculated by following formula [21].

$${Youden}^{\prime }s\;index=sensitivity+specificity-1$$

**Table 1:** Baseline characteristics of study population.

Variable	Value	Missing, n (%)
Demography		
Age, year	68 (56–78)	0 (0)
< 40, n (%)	1013 (7.2)	0 (0)
40–70, n (%)	6669 (47.6)	0 (0)
> 70, n (%)	6333 (45.2)	0 (0)
Sex (male), n (%)	8382 (59.8)	0 (0)
Category (disease), n (%)	12366 (88.2)	0 (0)
Suspected infection, n (%)	4054 (28.9)	0 (0)
Physiologic parameters		
Consciousness (alert), n (%)	11919 (85.0)	0 (0)
O_2_ supply, n (%)	5558 (39.7)	0 (0)
Systolic blood pressure, mmHg	120 (100–140)	45 (0.3)
Heart rate, per minute	88 (78–106)	41 (0.3)
Respiratory rate, per minute	20 (20–22)	41 (0.3)
Body temperature, °C	36.6 (36.3–37.1)	14 (0.1)
Oxyhemoglobin saturation, %	97 (94–98)	181 (1.3)
NEWS	6 (4–9)	209 (1.5)
Laboratory results		
Lactate, mmol/L	1.7 (1.1–3)	0 (0)
White blood cell, x10^3^/mm^3^	9.79 (6.89–13.86)	46 (0.3)
Hemoglobin, g/dl	12 (10.3–13.6)	46 (0.3)
Platelet, x10^3^/mm^3^	217 (159–281)	46 (0.3)
PTinr	1.09 (1.01–1.22)	68 (0.5)
Glucose, mg/dl	138 (112–189)	90 (0.6)
Creatinine, mg/dl	0.91 (0.69–1.31)	171 (1.2)
Bilirubin, mg/dl	0.61 (0.39–1.01)	75 (0.5)
C-reactive protein, mg/L	12.5 (1.7–74.1)	427 (3)
Base deficit, mEq/L	1.9 (-0.5–5.3)	903 (6.4)
30-day in hospital mortality, n (%)	1487 (10.6)	0 (0)

Abbreviations: NEWS, National Early Warning Score; PTinr, international normalized ratio of prothrombin time. Values are presented as median and interquartile range when not stated otherwise

Sensitivity, specificity, and positive and negative predictive values for the cutoff level were calculated. The derived lactate cutoff were assessed in subgroups of patients with disease or injury and patients with or without suspected infection. We divided patients into two groups (low-lactate and high-lactate group), and survival analysis was performed using the Kaplan-Meier method to obtain the 30-day survival curves of the groups. Differences between the groups were examined using the log-rank test. Because predictive values of a test vary depending on the prevalence of disease, we performed a sensitivity analysis to estimate the positive and negative predictive value based on plausible mortality rates.

The χ2 test was used to test for differences in categorical data. Independent *t*-test and Mann-Whitney *U* test were used for continuous data with normal and skewed distribution, respectively. All *p* values were two-sided, and a value of < 0.05 was considered statistically significant. Analyses were performed using MedCalc 17 (MedCalc Software BVBA, Ostend, Belgium) and Stata version 13 (StataCorp, LP, College Station, TX).

## Results

### Characteristics of study subjects

**Fig 1:**
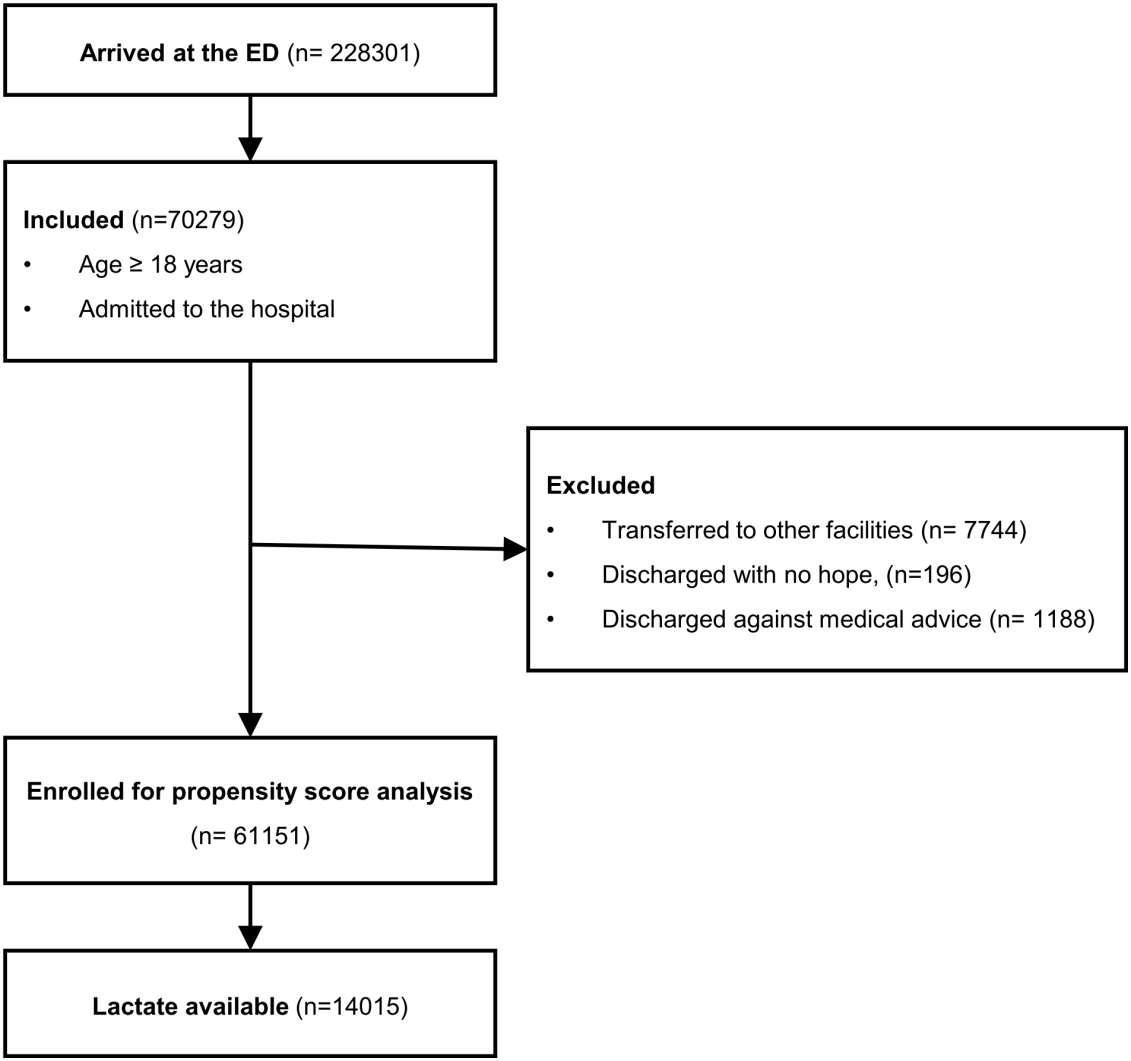
Inclusion and exclusion flow chart.

A total of 228,301 patients presented to the ED during the study period. 70,279 patients matched inclusion criteria (≥ 18 years of age, hospitalized after ED management). We excluded total 9,128 patients: 7,744 were transferred, 196 were discharged with no hope of recovery, and 1,188 were discharged against medical advice. After exclusion, 61,151 patients were used for propensity score analysis. Among them, serum lactate values were available in 14,015 patients that were used for final analysis (Fig 1). Age was categorized into three groups (<40, 40–70, and >70), and the NEWS was calculated in each patient. Basal characteristics of the patients are shown in Table 1.

### Propensity score analysis

In a multivariable logistic regression analysis, we selected statistically significant variables (*p* < 0.01) which explained the propensity of execution of the lactate test: age, consciousness, systolic blood pressure, breath rate, SpO_2_, WBC, hemoglobin, platelet, glucose, creatinine, bilirubin, CRP, and base deficit (Table 2). The ATE of carrying out a lactate test on 30-day in-hospital mortality was 0.53% (95% confidence interval (CI), -0.27%–1.32%), and not statistically significant (adjusted OR = 1.013, *p* = 0.19; 95% confidence interval (CI), 0.997–1.013).

**Table 2:** Adjusted odds ratio of variables on execution of lactate test.

Variable	Adjusted odds ratio (95% CI)	p value
Sex	0.937 (0.887–0.908)	0.011
Age	1.009 (1.008–1.031)	< 0.001
Consciousness (not alert)	2.376 (2.292–4.237)	< 0.001
Systolic blood pressure	0.987 (0.986–0.964)	< 0.001
Heart rate	1.002 (1.000–1.007)	0.016
Respiratory rate	1.051 (1.044–1.165)	< 0.001
Body temperature	1.000 (0.972–1.053)	0.992
Oxyhemoglobin saturation	0.983 (0.979–0.956)	< 0.001
White blood cell	1.005 (1.002–1.021)	0.001
Hemoglobin	0.968 (0.957–0.925)	< 0.001
Platelet	0.999 (0.999–0.999)	< 0.001
PTinr	1.002 (0.974–1.063)	0.871
Glucose	1.002 (1.001–1.006)	< 0.001
Creatinine	1.043 (1.028–1.156)	< 0.001
Bilirubin	0.958 (0.946–0.896)	< 0.001
C-reactive protein	0.998 (0.998–0.996)	< 0.001
Base deficit	1.010 (1.005–1.041)	< 0.001

### Prognostic properties of serum lactate in unselected patients and subgroups

Univariable and multivariable logistic regression analyses were performed on multiply imputed datasets. All the variables (age, sex, NEWS, WBC, hemoglobin, platelet, PTinr, creatinine, bilirubin, CRP, base deficit, and lactate) were significant in univariable analysis (Table 3), and the adjusted OR of serum lactate calculated from multivariable analysis was 1.09 (*p* < 0.001; 95% CI, 1.07–1.10) (Table 4). The area under a ROC curve (AUC) of serum lactate was 0.711 (*p* < 0.001; 95% CI, 0.703–0.718). The sensitivity, specificity, and positive and negative predictive values for the cutoff > 2.6 mmol/L (the point of maximum Youden’s index) were 56.7%, 74.3%, 20.8%, and 93.5%, respectively (Table 5).

**Table 3:** Results of univariable analysis on multiply imputed datasets.

Variable	Odds ratio (95% CI)	p value
Age	1.0150 (1.0112–1.0188)	< 0.001
<40	-	-
40–70	2.8415 (2.0424–3.9531)	< 0.001
>70	3.4120 (2.4566–4.7389)	< 0.001
Sex (female)	0.7010 (0.6255–0.7856)	< 0.001
Lactate	1.1546 (1.1389–1.1704)	< 0.001
White blood cell	1.0318 (1.0246–1.0391)	< 0.001
Hemoglobin	0.9068 (0.8886–0.9254)	< 0.001
Platelet	0.9981 (0.9975–0.9986)	< 0.001
PTinr	1.2496 (1.1982–1.3032)	< 0.001
Glucose	1.0011 (1.0006–1.0015)	< 0.001
Creatinine	1.0869 (1.0619–1.1124)	< 0.001
Bilirubin	1.1433 (1.1226–1.1643)	< 0.001
C-reactive protein	1.0050 (1.0045–1.0056)	< 0.001
Base deficit	1.0853 (1.0771–1.0935)	< 0.001
NEWS	1.1877 (1.1703–1.2053)	< 0.001

**Table 4:** Adjusted odds ratios of serum lactate in multivariable analysis.

Category	n	Odds ratio (95% CI)	*p* value
All patients	14015	1.09 (1.07–1.10)	0.000
Patients with disease	12366	1.10 (1.08–1.12)	0.000
Patients with injury	1649	1.05 (1.03–1.08)	0.000
Patients with suspected infection	9961	1.08 (1.06–1.10)	0.000
Patients without suspected infection	4054	1.09 (1.06–1.12)	0.000

Abbreviations: CI, confidence interval.

**Table 5:** Prognostic properties of serum lactate (cutoff > 2.6 mmol/L).

Category	Sensitivity	Specificity	PPV	NPV
	(95% CI)	(95% CI)	(95% CI)	(95% CI)
All patients	56.7	74.3	20.8	93.5
	(54.1–59.2)	(73.5–75.1)	(19.9–21.6)	(93.2–93.9)
Patients with disease	54.7	76.5	22.1	93.3
	(52.0–57.4)	(75.7–77.3)	(21.1–23.1)	(92.9–93.7)
Patients with injury	74.8	58.2	14.9	95.9
	(67.0–81.6)	(55.6–60.7)	(13.5–16.4)	(94.7–96.9)
Patients with suspected infection	58.2	76.6	19.9	94.8
	(54.9–61.4)	(75.7–77.5)	(18.8–21.0)	(94.5–95.2)
Patients without suspected infection	54.4	68.2	22.4	89.9
	(50.2–58.4)	(66.7–69.8)	(20.9–24.0)	(89.0–90.7)

Abbreviations: CI, confidence interval; PPV, positive predictive value; NPV, negative predictive value

Adjusted OR of lactate in subgroups were all statistically significant (*p* < 0.001) (Table 4). We chose the cutoff > 2.6 mmol/L for subgroups analysis (patients with disease or injury, and patients with or without suspected infection). Prognostic properties of lactate in subgroups were summarized in Table 5.

### Survival analysis

**Fig 2:**
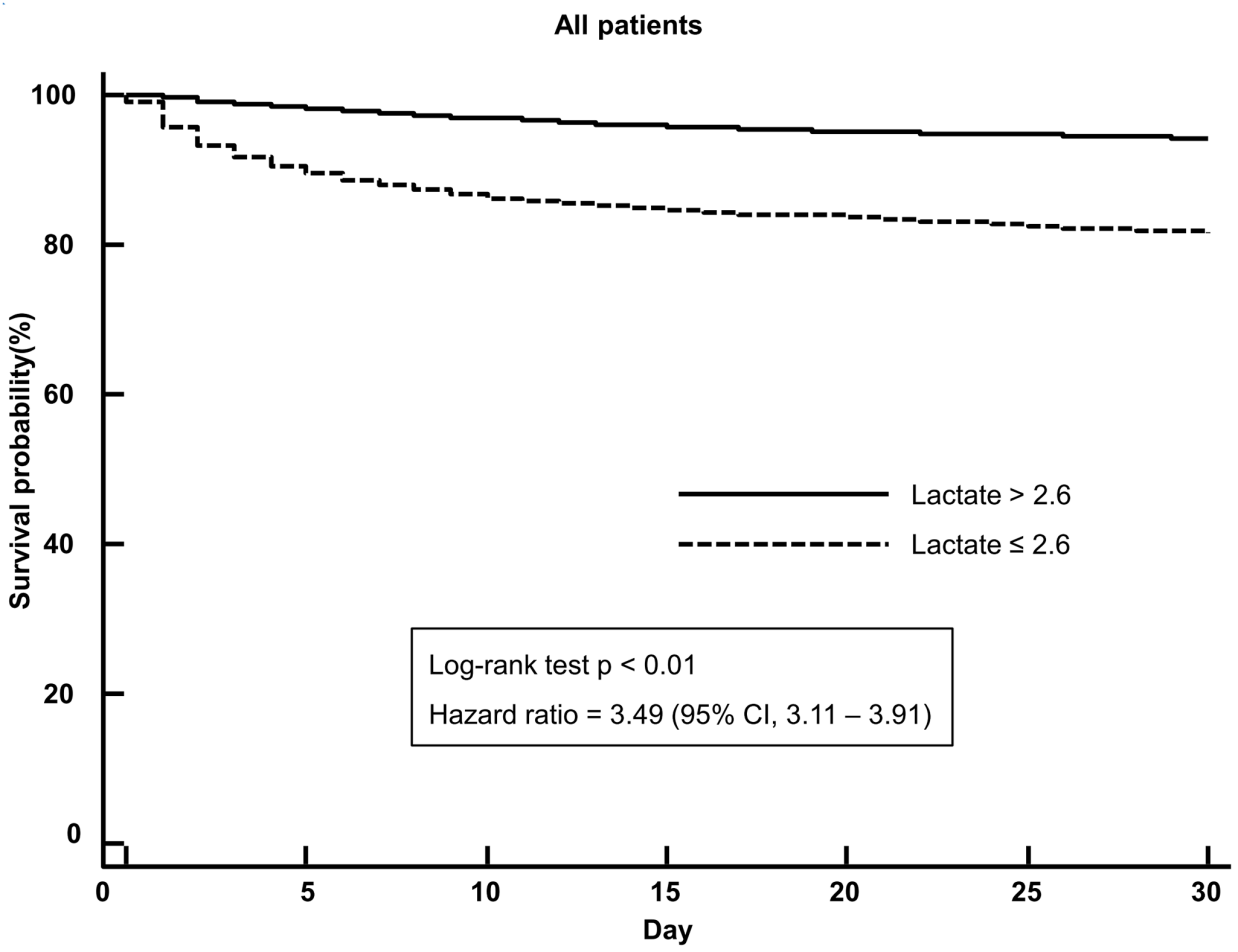
Kaplan-Meier survival curves for 30-day survival according to the cutoff (>2.6 mmol/L) in all patients.

**Fig 3:**
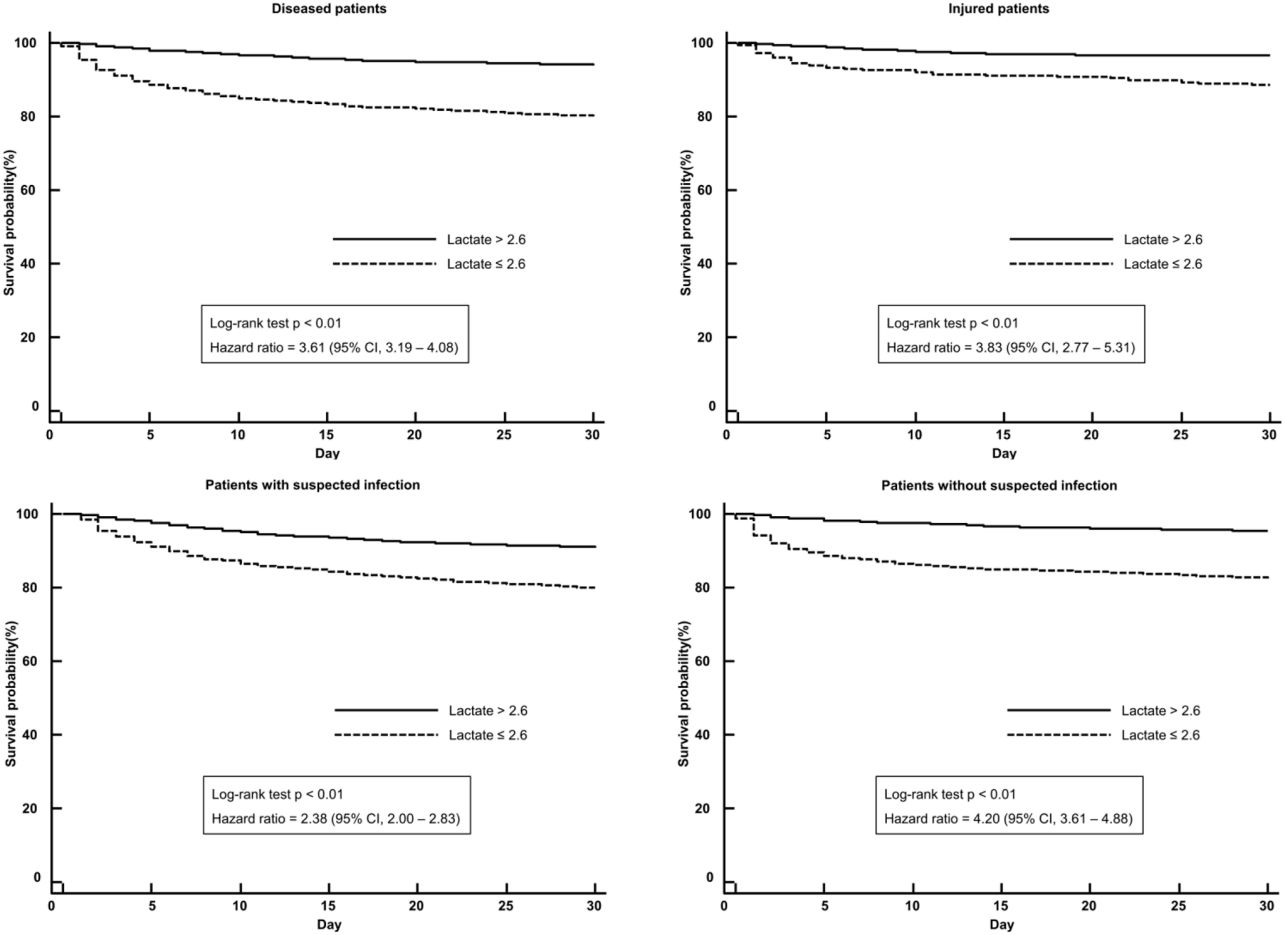
Kaplan-Meier survival curves for 30-day survival according to the cutoff (>2.6 mmol/L) in subpopulations.

Mortality of the high-lactate group (> 2.6 mmol/L) was significantly higher than that of the low lactate group (≤ 2.6 mmol/L) (20.8% vs. 6.5%, difference = 14.3%, *p* < 0.01; 95% CI, 13.0%–15.7%). In patients with medical illness (n = 12,366), the high-lactate group showed significantly higher mortality than the low-lactate group (22.1% vs. 6.7%, difference = 15.4%, *p* < 0.01; 95% CI, 13.9%–16.9%). In patients with injury (n = 1,649), the high-lactate group showed significantly higher mortality than the low-lactate group (14.9% vs. 4.1%, difference = 10.8%, *p* < 0.01; 95% CI, 7.9%–13.8%). In patients with suspected infection (n = 4,054), the high-lactate group showed significantly higher mortality than the low-lactate group (22.4% vs. 10.1%, difference = 12.3%, *p* < 0.01; 95% CI, 9.8%–14.8%). In patients without suspected infection (n = 9,961), the high-lactate group showed significantly higher mortality than the low-lactate group (19.9% vs. 5.2%, difference = 14.7%, *p* < 0.01; 95% CI, 13.1%–16.4%). Kaplan-Meier curves for unselected patients and subgroups were illustrated in Figs 2 and 3.

### Sensitivity analysis

Mortality rate of our study population was 10.6%, and those of subgroups were ranged from 8.9 to 14.4%. Because predictive values of serum lactate varied depending on mortality rate in our study, we performed a sensitivity analysis for the positive and negative predictive value based on plausible mortality rates ranged from 1 to 40% (Table 6).

**Table 6:** Sensitivity analysis for predictive values.

Category	PPV (95% CI)	NPV (95% CI)	Mortality (%)
All patients			
	59.5 (58.2–60.8)	72.0 (70.8–73.2)	40
	48.6 (47.3–49.9)	80.0 (79.1–80.9)	30
	35.6 (34.3–36.8)	87.3 (86.6–87.9)	20
	28.0 (27.0–29.1)	90.7 (90.2–91.2)	15
	20.8 (19.9–21.6)	93.5 (93.2–93.9)	10.6^a^
	10.4 (9.9–10.9)	97.0 (96.8–97.2)	5
	6.4 (6.1–6.7)	98.2 (98.1–98.3)	3
	2.2 (2.1–2.3)	99.4 (99.4–99.4)	1
Patients with disease			
	60.8 (59.4–62.2)	71.7 (70.5–72.9)	40
	49.9 (48.5–51.4)	79.8 (78.8–80.7)	30
	36.8 (35.4–38.2)	87.1 (86.4–87.8)	20
	29.1 (27.9–30.4)	90.5 (90.0–91.0)	15
	22.1 (21.1–23.1)	93.3 (92.9–93.7)	10.84^a^
	10.9 (10.4–11.5)	97.0 (96.8–97.1)	5
	6.7 (6.4–7.1)	98.2 (98.1–98.3)	3
	2.3 (2.2–2.4)	99.4 (99.4–99.4)	1
Patients with injury			
	54.4 (51.6–57.1)	77.6 (72.3–82.1)	40
	43.4 (40.7–46.2)	84.4 (80.3–87.7)	30
	30.9 (28.6–33.3)	90.2 (87.5–92.5)	20
	24.0 (22.0–26.1)	92.9 (90.8–94.6)	15
	14.9 (13.5–16.4)	95.9 (94.7–96.9)	8.9^a^
	8.6 (7.8–9.5)	97.8 (97.1–98.3)	5
	5.2 (4.7–5.8)	98.7 (98.3–99.0)	3
	1.8 (1.6–2.0)	99.6 (99.4–99.7)	1
Patients with suspected infection			
	53.3 (51.1–55.5)	69.2 (67.2–71.1)	40
	42.3 (40.2–44.5)	77.7 (76.1–79.3)	30
	30.0 (28.1–31.9)	85.7 (84.5–86.8)	20
	22.4 (20.9–24.0)	89.9 (89.0–90.7)	14.43^a^
	16.0 (14.8–17.2)	93.1 (92.5–93.6)	10
	8.3 (7.6–9.0)	96.6 (96.3–96.9)	5
	5.0 (4.6–5.5)	98.0 (97.8–98.1)	3
	1.7 (1.6–1.9)	99.3 (99.3–99.4)	1
Patients without suspected infection			
	62.4 (60.8–64.0)	73.3 (71.8–74.8)	40
	51.6 (50.0–53.3)	81.1 (79.8–82.2)	30
	38.4 (36.8–40.0)	88.0 (87.2–88.8)	20
	30.5 (29.1–32.0)	91.2 (90.6–91.8)	15
	19.9 (18.8–21.0)	94.8 (94.5–95.2)	9.06^a^
	11.6 (10.9–12.3)	97.2 (97.0–97.4)	5
	7.2 (6.7–7.6)	98.3 (98.2–98.5)	3
	2.5 (2.3–2.6)	99.5 (99.4–99.5)	1

^a^ Observed mortality in study population

## Discussion

In this study, the AUC of initial lactate level as a predictor of 30-day in-hospital mortality in unselected patients who presented at the ED was 0.711. The discrimination power was acceptable according to the standard suggested by Metz CE (0.9–1 excellent, 0.8–0.9 good, 0.7–0.8 fair, 0.6–0.7 poor, 0.5–0.6 fail) [22]. Statistical significance of the cutoff of lactate > 2.6 mmol/L was maintained even after categorization of the patients into groups according to etiology (disease and injury) and the presence of suspected infection. Higher mortality in ED patients with mild hyperlactatemia has been addressed in many previous studies. In a systematic review of the prognosis of ED patients with suspected infection, lower lactate levels (2.0–4.0 mmol/L) were associated with higher mortality [6]. Several studies showed consistent results in trauma patients [23, 24]. In these studies, cutoffs of serum lactate associated with higher mortality were 2.5–4.0 mmol/L. There have been very few studies in unselected ED patients on this issue. In a retrospective cohort study involving 5,360 ED patients, intermediate lactate levels (2.0–3.9 mmol/L) were shown to be associated with a worse outcome [7]. A prospective cohort study involving 747 ill ED patients showed that initial mild hyperlactatemia (2.0–4.0 mmol/L) was associated with higher mortality [8]. Despite numerous studies showed that mild hyperlactatemia (cutoffs ranged from 2.0 to 2.5) was associated with unfavorable outcome, there have been no studies demonstrating optimal cutoffs of serum lactate. To our best knowledge, this is the first study indicating a lactate cutoff value for detection of high risk patients among unselected ED population.

We performed a propensity score analysis in order to estimate selection bias and to justify that our study population was unselected. The ATE of carrying out lactate test on 30-day in-hospital mortality was only 0.53% and statistically insignificant. This means, in our study, execution of lactate test was not associated with patient outcome, and therefore selection bias from including patients who underwent lactate test only was minimal.

The sensitivity and specificity of serum lactate were 56.7% and 74.3. At the cutoff of lactate > 2.6 mmol/L, positive and negative predictive value were 20.8%, and 93.5%. This tendency of prognostic properties were consistently shown in the study by Filho et al. In the study, sensitivity, specificity, and positive and negative predictive values were 67.4%, 61.7%, 16.9%, and 94.2%, respectively, and AUC of serum lactate was 0.70 [11]. The authors demonstrated a cutoff of > 2.5 mmol/L which was similar to our results. The low positive predictive value and high negative predictive value indicate that serum lactate test might be an effective screening tool. We believe these consistent results from different population (ICU patients with sepsis and unselected ED patients) imply that serum lactate test should be more widely used for early detection of high risk patients in various clinical settings.

Overall mortality in this study (10.6%) was probably higher than general population presented in the ED because we only included patients admitted after ED management. Sensitivity analysis showed rule-out capacity of blood lactate test could be improved with mortality decrease in target population. For example, in a certain emergency department where an overall mortality rate is 1% (lower than our population, 10.6% in all patients), estimated positive and negative predictive value of lactate > 2.6 mmol/L are 2.2% and 99.4% (i.e., only 0.6% patients of ≤ lactate 2.6 mmol/L will die), lactate test in such population would be an excellent screening tool for discriminating low risk patients.

One limitation to the implementation of routine lactate test is the cost. Ward MJ et al. reported point-of-care lactate testing for screening ED patients with suspected sepsis is cost-effective to identify patients responsive to early resuscitation [25]. However, there have been no studies on the cost-effectiveness of lactate test for screening in unselected ED population. In addition, hyperlactatemia is not just caused by real serious conditions with sustained tissue hypoxia (type A hyperlactatemia) including type B hyperlactatemia such as brief seizure, asthmatic attack, drugs, various chronic diseases, and inborn errors of metabolism. Therefore, lactate level should be used as a part of decision making with consideration in clinical presentation and other diagnostic tools.

Neverthless, we believe that routine blood lactate test in unselected patients may play an important role as a screening tool in the ED. It could alleviate ED overcrowding by fast decision making on patients with lower risk of poor prognosis, and could contribute to reduce adverse events such as ED revisit and early death after discharge.

This study has several limitations. First, it is known that the propensity score analysis does not correct for all selection biases; thus, potential biases in this study existed. Second, information about comorbidity that could influence the propensity to execute a serum lactate test was not collected. However, the physician’s decision to order a lactate test in the ED was more dependent on the patients’ current condition, not the underlying status. Third, although arterial blood was collected for the lactate test in most cases, we could not identify whether a given result was from arterial or venous blood. However, current guidelines state that either collection is appropriate for the management of sepsis and septic shock [10]. Fourth, Vasilevskis et al. stated that 30-day in-hospital mortality could be biased by the proportion of transferred patients and early post-discharge mortality [26]. Transferred patients were excluded from our analysis, whereas we could not identify patients who died after discharge in our study.

## Conclusion

In our retrospective cohort study, a serum lactate level > 2.6 mmol/L predicted a 30-day in-hospital mortality in unselected patients who arrived at the ED and were admitted to the hospital. Additionally, serum lactate test in the ED could be an effective screening method for identifying low risk patients.
